# Association of muscular fitness with rehospitalization for heart failure with reduced ejection fraction

**DOI:** 10.1002/clc.23535

**Published:** 2020-12-25

**Authors:** Chan Joo Lee, Ho Youl Ryu, Kyeong‐Hyeon Chun, Jaewon Oh, Sungha Park, Sang‐Hak Lee, Seok‐Min Kang

**Affiliations:** ^1^ Division of Cardiology, Department of Internal Medicine, Severance Hospital Yonsei University College of Medicine Seoul Republic of Korea

**Keywords:** heart failure, muscle power, muscle strength, rehospitalization

## Abstract

**Background:**

Limited information is available regarding the prognostic potential of muscular fitness parameters in heart failure (HF) with reduced ejection fraction (HFrEF).

**Hypothesis:**

We aimed to investigate the predictive potential of knee extensor muscle strength and power on rehospitalization and evaluate the correlation between exercise capacity and muscular fitness in patients newly diagnosed with HFrEF.

**Methods:**

Ninety nine patients hospitalized with a new diagnosis of HF were recruited (64 men; aged 58.7 years [standard deviation (SD), 13.2 years]; 32.3% ischemic; ejection fraction, 28% [SD, 8%]). The inclusion criteria were left ventricular ejection fraction <40% and sufficient clinical stability to undergo exercise testing. Aerobic exercise capacity was measured with cardiopulmonary exercise testing. Knee extensor maximal voluntary isometric contraction (MVIC) and muscle power (MP) were measured using the Baltimore therapeutic equipment system. The clinical outcome was HF rehospitalization.

**Results:**

Over a mean follow‐up period of 1709 ± 502 days, 39 patients were rehospitalized due to HF exacerbation. HF rehospitalization was more probable for patients with diabetes and lower oxygen uptake at peak exercise (peak VO_2_), knee extensor MVIC, and MP. The Kaplan–Meier survival analysis revealed significantly different cumulative HF rehospitalization rates according to the tertiles of peak VO_2_ (*P* = 0.005) and MP (*P* = 0.002). Multivariable Cox proportional hazard model showed that the lowest tertiles of peak VO_2_ (hazard ratio (HR), 6.26; 95% confidence interval (CI), 1.93–20.27); and MP (HR, 5.29; 95% CI, 1.05–26.53) were associated with HF rehospitalization. Knee extensor muscle power was an independent predictor for rehospitalization in patients with HFrEF.

**Conclusion:**

Knee extensor muscle power was an independent predictor for rehospitalization in patients with HFrEF.

## 1 INTRODUCTION

Heart failure (HF) is a chronic debilitating disease associated with a 50% mortality rate within 5 years of diagnosis.^1^ Despite advancements in treatment strategies, HF still poses a significant threat to patient outcomes.^2^ Patients with HF often complain of fatigue and shortness of breath even with low‐intensity physical activity, which causes detrimental effects to their quality of life.^3^, ^4^ Exercise intolerance is a consequence of HF and a major determinant of its prognosis.^5^, ^6^ Measurement of peak oxygen uptake (peak VO_2_) during exercise is an objective method for assessing functional capacity and is an important predictor of long‐term prognosis in patients with HF.^7^ Peak VO_2_ is used for determining the timing of heart transplantation.^8^

The loss of muscle mass and strength occurs progressively with aging.^9^ Irrespective of aging, chronic diseases accelerate the atrophy of muscle fibers or lower the efficiency of energy production in the muscles, leading to low levels of muscular fitness, which is also associated with poor prognosis.^10^, ^11^ In patients with HF, muscular strength predict long‐term survival.^6^ However, the effect of muscle fitness on rehospitalization has not been fully investigated. Rehospitalization is an important outcome for patients with HFrEF, as it relates to quality of life and high financial burdens for the community.^12^ Muscular strength is one of the most common indicators of muscular fitness. However, muscle performance can also be measured as muscle power (MP), which indicates the ability of the muscle to perform forceful and high‐velocity movements. Furthermore, as the measurement of MP is technically simple and relatively easy to assess, it could be incorporated into a standard prognostic assessment for patients with HFrEF. Nevertheless, indices of muscular fitness are rarely used in real‐world clinical practice, unlike peak VO_2_.

Therefore, we aimed to do the following: (a) investigate the predictive potential of knee extensor muscle strength and power on rehospitalization; and (b) evaluate the correlation between exercise capacity and muscular fitness in patients newly diagnosed with HFrEF.

## 2 MATERIAL AND METHODS

### 2.1 Study participants

We recruited consecutively a total of 99 patients, who were hospitalized with a new diagnosis of HF from January 2013 to November 2015, received subsequent inpatient treatment, and were discharged. The inclusion criteria were as follows: left ventricular ejection fraction <40% and sufficient clinical stability to undergo exercise testing. Patients who underwent surgical procedures such as coronary artery bypass, valve replacement, heart transplantation, and those on renal replacement therapy were excluded. Aerobic exercise capacity and muscular fitness were examined immediately before discharge. We investigated the medications prescribed at discharge and obtained blood chemistry data at the time of the 1–2 week follow‐up visit. Definition of heart failure rehospitalization was a hospitalization caused by worsening heart failure symptoms and signs requiring the augmentation of previous medications.^13^ We identified cases of heart failure rehospitalization by chart reviewing. All patients provided written informed consent at enrollment, and the Ethics Committee of Severance Hospital of the Yonsei University Health System approved the protocol (No. 4–2018‐1180). The study was performed in accordance with the Declaration of Helsinki.

### 2.2 Assessment of aerobic exercise capacity

Functional exercise capacity was evaluated during the maximal treadmill exercise test using the Bruce RAMP protocol with the cardiopulmonary exercise test (CPET) system CASE T2100 (GE Healthcare, Chicago, IL) under the supervision of a cardiologist. Respiratory gas exchange analysis was performed throughout the exercise protocol with a Quark gas analysis system (COSMED, Rome, Italy).

### 2.3 Assessment of muscular fitness

The assessments of two muscular fitness parameters were performed using the Primus RS, version 11 (Baltimore Therapeutic Equipment Technology, Hanover, MD). For knee extensor muscle strength, the maximal voluntary isometric contraction (MVIC) and MP were measured. For measurements of MVIC (Supplemental Video File 1), participants were instructed to push with maximum force while keeping the knee flexed at 45°, and the mean value of three measurements was obtained. To compensate for differences in body weight among participants, the value was divided into MVIC per kg of body weight for use in the statistical analysis. For the assessment of MP (Supplemental Video File 2), resistance corresponding to 20% of body weight was applied to compensate for differences in body weight between the participants, after which, participants were instructed to flex and extend the knees with maximum effort. The mean value of the top five results from 10 measurements was used in the statistical analysis.

### 2.4 Statistical analysis

Data were expressed as mean ± standard deviation (SD) frequency (%), or median (interquartile range [IQR]). Patients were grouped based on whether they required HF rehospitalization. For group comparison of continuous variables, the Student's t‐test, one‐way analysis of variance, the Mann–Whitney U test, or the Kruskal‐Wallis test was used. Categorical variables were evaluated using chi‐squared test or Fisher's exact test. Correlation analysis between aerobic exercise capacity and muscular fitness was performed using Pearson's correlation coefficient. The effect of aerobic exercise capacity or muscular fitness on HF rehospitalization was analyzed using Kaplan–Meier curves. Receiver operating characteristic (ROC) analysis was performed to identify the best cut‐off value of peak VO_2_, MVIC, and MP for HF rehospitalization. The association of the tertile of aerobic exercise capacity or muscular fitness with HF rehospitalization was evaluated using a multivariable Cox proportional hazard model with adjustment for age, sex, body mass index, diabetes mellitus, and left ventricular ejection fraction (LVEF), and N‐terminal pro‐B‐type natriuretic peptide (NT‐proBNP). Statistical analyses were conducted using R software, version 3.5.3 (R Foundation for Statistical Computing, Vienna, Austria), assuming a threshold of significance at *P* < 0.05.

## 3 RESULTS

### 3.1 Baseline characteristics of patients

Table 1 shows the baseline characteristics of the patients. The mean age was 58.7 years (SD, 13.2 years), and 64 patients (66.3%) were male. Their left ventricular (LV) systolic function was markedly impaired (mean LVEF, 27.5% [SD, 8.1%]). Ischemic cardiomyopathy accounted for 32.3% of the etiologies of HF.

**TABLE 1:** Baseline characteristics according to rehospitalization in total subjects

	Total (*N* = 99)	No rehospitalization (*N* = 60)	Rehospitalization (*N* = 39)	*P* value	
*Demographic findings*					
Age, years	58.3 SD, 13.3	57.3 SD, 12.2	59.9 SD, 15.0	0.351	
Men, N(%)	64 (64.6%)	43 (71.7%)	21 (53.8%)	0.110	
Height, cm	163.9 ± 8.9	164.6 ± 9.0	162.7 ± 8.6	0.276	
Weight, kg	64.4 ± 14.7	65.7 ± 13.0	62.4 ± 16.9	0.274	
BMI, kg/m^2^	23.8 ± 4.1	24.1 ± 3.6	23.4 ± 4.8	0.427	
LV ejection fraction, %	27.5 ± 8.1	29.1 ± 8.4	25.1 ± 7.3	0.018	
Sinus rhythm, N(%)	73 (73.7%)	44 (73.3%)	29 (74.4%)	0.999	
DM, N(%)	25 (27.8%)	9 (17.3%)	16 (42.1%)	0.018	
Hypertension, N(%)	39 (43.3%)	21 (40.4%)	18 (47.4%)	0.656	
Ischemic cardiomyopathy, N(%)	32 (32.3%)	22 (36.7%)	10 (25.6%)	0.354	
*Laboratory findings*					
BUN, mg/dl	18.5 ± 6.6	18.4 ± 5.9	18.8 ± 7.7	0.769	
Creatinine, mg/dl	0.9 ± 0.4	1.0 ± 0.2	0.9 ± 0.5	0.390	
Serum total protein, d/dl	6.7 ± 0.6	6.8 ± 0.7	6.7 ± 0.6	0.662	
Serum albumin, d/dl	4.0 ± 0.5	4.0 ± 0.5	3.9 ± 0.4	0.559	
Na^+^, mmol/L	140.0 ± 2.6	140.4 ± 2.4	139.4 ± 2.9	0.071	
K^+^, mmol/L	4.4 ± 0.5	4.5 ± 0.5	4.3 ± 0.4	0.078	
NT‐proBNP, pg/ml	1389.5 (718.0–2248.0)	1429.5 (680.0–2263.0)	1342.5 (817.0–1943.5)	0.840	
*Medications at discharge*					
ACE inhibitor, N(%)	42 (42.4%)	24 (40.0%)	18 (46.2%)	0.691	
ARB, N(%)	36 (36.4%)	23 (38.3%)	13 (33.3%)	0.771	
Beta blocker, N(%)	75 (75.8%)	52 (86.7%)	23 (59.0%)	0.004	
Ivabradine, N(%)	10 (10.1%)	5 (8.3%)	5 (12.8%)	0.702	
Loop diuretics, N(%)	74 (74.7%)	43 (71.7%)	31 (79.5%)	0.523	
MRA, N(%)	74 (74.7%)	45 (75.0%)	29 (74.4%)	0.999	

*Note*: Data are presented as mean ± SD, N(%) or median (IQR).

Abbreviations: ACE, angiotensin converting enzyme; ARB, angiotensin receptor blocker; BMI, body mass index; BUN, blood urea nitrogen; DM, diabetes mellitus; LV, left ventricular; CMP; MRA, mineralocorticoid antagonist; NT‐proBNP. N‐terminal pro b‐type natriuretic peptide; SD, standard deviation.

During the follow‐up period (mean: 1691 days [SD, 512 days]; median: 1762 days [IQR, 1588–2059 days]), 39 patients (39.4%) were rehospitalized due to HF aggravation. Patients with rehospitalization had lower LVEF and a higher rate of diabetes than those without HF rehospitalization. Regarding HF guideline‐directed medications at baseline, the use of beta‐blockers was significantly lower in patients with HF rehospitalization than in those without HF rehospitalization. The use of other medications was not significantly different between the two groups.

Table 2 shows the results of CPET and muscular fitness tests. Patients with rehospitalization had significantly lower heart rates at rest and maximal exercise in CPET. The systolic blood pressure did not differ between the two groups at rest; however, it was lower during maximal exercise in patients with rehospitalization. Moreover, the exercise time and peak VO_2_ were significantly lower in patients with rehospitalization, indicating a clear difference in aerobic exercise performance between the two groups. The MVIC was significantly lower in patients with rehospitalization. The MP also tended to be lower in patients with HF rehospitalization. These results showed that the patients with HF rehospitalization presented with lower values for skeletal muscle fitness parameters than those without rehospitalization.

**TABLE 2:** Parameters of cardiopulmonary exercise test and muscle fitness measurement according to rehospitalization in total subjects

	Total	No rehospitalization	Rehospitalization	*P* value
	(*N* = 99)	(*N* = 60)	(*N* = 39)	
*Cardiopulmonary exercise test*				
Heart rate at rest, bpm	84.3 ± 17.6	87.4 ± 18.3	79.5 ± 15.3	0.028
SBP at rest, mmHg	106.8 ± 17.9	108.7 ± 19.6	103.8 ± 14.8	0.187
Peak VO_2_, ml/kg/min	20.0 ± 5.6	21.6 ± 5.6	17.7 ± 4.6	<0.001
Exercise time, sec	546.3 ± 196.7	604.2 ± 184.8	457.2 ± 182.6	<0.001
AT, ml/kg/min	15.7 ± 5.7	16.4 ± 5.8	14.7 ± 5.6	0.161
RER at peak	1.1 ± 0.1	1.1 ± 0.1	1.1 ± 0.1	0.273
VE/VCO_2_ slope	36.8 ± 8.2	35.7 ± 7.9	38.4 ± 8.5	0.115
PetCO_2_, mmHg	33.5 ± 6.7	33.7 ± 5.5	33.2 ± 8.4	0.746
HR at maximal exercise, bpm	137.5 ± 28.3	145.1 ± 30.7	125.9 ± 19.5	<0.001
HR reserve, bpm	53.3 ± 22.8	57.8 ± 24.9	46.4 ± 17.3	0.009
SBP at maximal exercise, mmHg	150.1 ± 30.7	157.4 ± 31.3	139.0 ± 26.3	0.003
SBP reserve, mmHg	43.4 ± 23.6	48.7 ± 24.8	35.2 ± 19.3	0.005
HRR/SBPR	1.6 ± 1.6	1.6 ± 1.8	1.7 ± 1.1	0.651
*Muscle fitness measurement*				
MVIC (N)	373.3 ± 138.6	399.6 ± 131.4	332.9 ± 141.2	0.018
MP (Watt)	134.1 ± 74.3	145.7 ± 67.5	116.2 ± 81.3	0.052

Abbreviations: AT, anaerobic threshold; HR, heart rate; HRR, heart rate reserve; MP, muscle power; MVIC, maximum voluntary isomeric contraction; PetCO_2,_ pulmonary end‐tidal CO_2;_ RER, respiratory exercise ratio; SBP, systolic blood pressure; SBPR, systolic blood pressure reserve; VCO_2_, carbon dioxide production; VE, ventilatory equivalents; VO_2_, oxygen uptake.

### 3.2 Correlations among parameters of exercise capacity and muscular fitness

**FIGURE 1:**
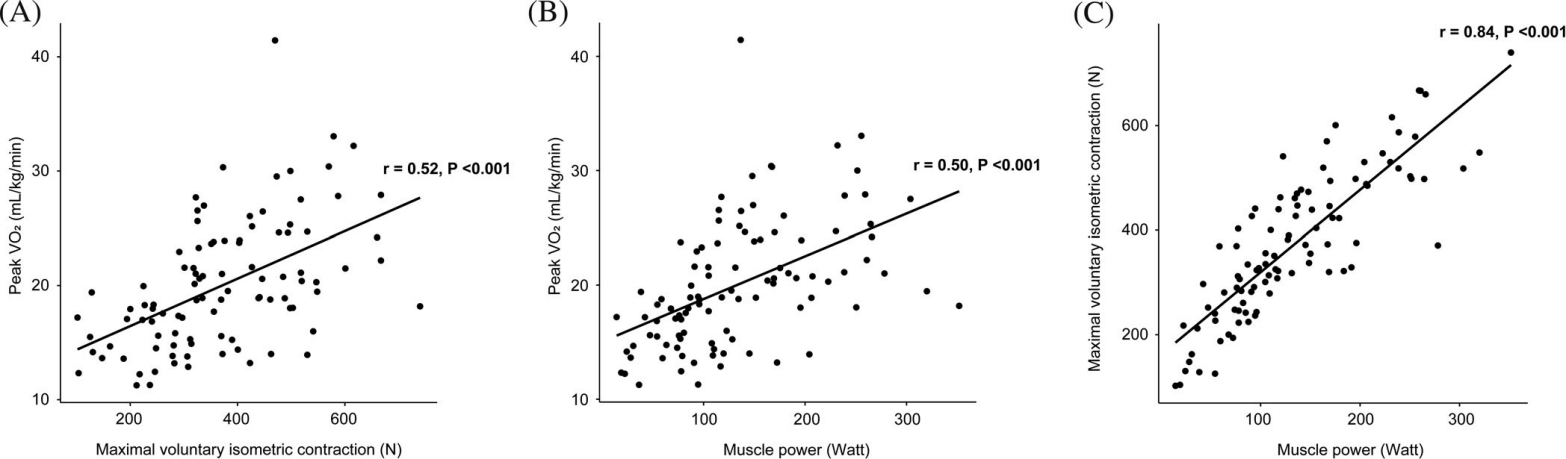
Scatterplots and correlation between (A) aerobic exercise capacity and maximal voluntary isometric contraction, (B) aerobic exercise capacity and muscle power, and (C) maximal voluntary isometric contraction and muscle power. VO_2_, oxygen uptake

Patients with higher muscular fitness exhibited higher aerobic exercise capacity (Supplemental Figure 1). MVIC (r = 0.52; 95% confidence interval (CI), 0.36–0.65; *P* < 0.001) and MP (r = 0.50; 95% CI, 0.34–0.63; *P* < 0.001) showed good correlation with peak VO_2_ (Figure 1(A),(B)). In addition, MVIC and MP showed high correlation (r = 0.84; 95% CI, 0.78–0.89; *P* < 0.001; Figure 1(C)).

### 3.3 Muscular fitness as a predictor for rehospitalization

**FIGURE 2:**
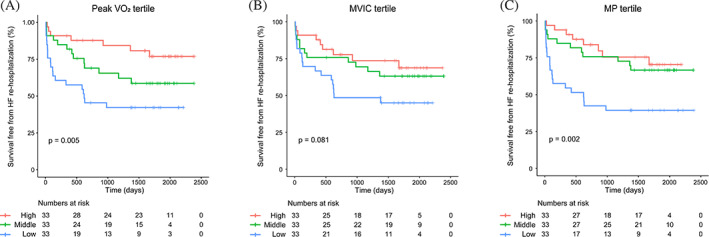
Kaplan–Meier curves for heart failure (HF) rehospitalization according to the tertile of (A) aerobic exercise capacity, (B) maximal voluntary isometric contraction (MVIC), and (C) muscle power (MP). VO_2_, oxygen uptake

Supplemental Table 1 shows the incidence of HF rehospitalization during the follow‐up period according to the peak VO_2_, MVIC, and MP tertiles. The incidence of HF rehospitalization was significantly different according to the peak VO_2_ tertile. Patients were divided into three groups (low, middle, and high) according to the tertiles of peak VO_2_, MVIC, and MP. Patients with lower aerobic exercise capacity had more HF rehospitalizations during follow‐up (Figure 2). Patients with lower MVIC tended to present with HF rehospitalization more frequently; however, the difference was not statistically significant (Supplemental Table 1), as was the case with the Kaplan–Meier survival curve (Figure 2). Nevertheless, patients with low MP had significantly more HF rehospitalizations than the other groups (Supplemental Table 1). The Kaplan–Meier curve showed that patients with low MP were rehospitalized for HF at early periods of follow‐up (Figure 2). ROC curves showed that the best cut‐off values of peak VO_2_, MVIC, and MP for HF rehospitalization was 20.1 ml/kg, 320 N, and 87 Watt, respectively (Supplemental Figure 2). The best cut‐off value of peak VO_2_ was similar to the high tertile value, while the best cut‐off values of MVIC and MP were similar to the low tertile values.

In the multivariable Cox proportional hazard model (Table 3), low LVEF and DM were important predictors for HF rehospitalization. Patients with low peak VO_2_ had a significantly higher risk of HF rehospitalization than those with high peak VO_2_ (hazard ratio, 6.26; 95% CI, 1.93–20.27; *P* = 0.002). Of the indicators for muscular fitness, only MP showed a significant association with HF rehospitalization. The patients in the low MP group were 5.29‐times more apt to be rehospitalized for HF than those in the high MP group.

**TABLE 3:** Multivariable Cox proportional hazard regression analyses for HF rehospitalization according to the tertiles of aerobic exercise capacity and muscle fitness

	HR (95% CI)	*P* value		HR (95% CI)	*P* value		HR (95% CI)	*P* value
Age	1.00 (0.97–1.02)	0.733	Age	0.99 (0.97–1.02)	0.692	Age	0.99 (0.95–1.03)	0.540
Female	1.18 (0.54–2.55)	0.681	Female	1.31 (0.54–3.22)	0.551	Female	0.97 (0.36–2.60)	0.945
BMI	1.01 (0.91–1.11)	0.897	BMI	1.01 (0.90–1.13)	0.869	BMI	1.04 (0.84–1.15)	0.431
DM	2.96 (1.50–5.85)	0.002	DM	3.54 (1.69–7.42)	<0.001	DM	2.64 (1.34–5.21)	0.005
LVEF	0.93 (0.88–0.97)	0.001	LVEF	0.92 (0.88–0.97)	0.001	LVEF	0.93 (0.69–0.97)	0.002
NT‐proBNP	1.00 (1.00–1.00)	0.837	NT‐proBNP	1.00 (1.00–1.00)	0.831	NT‐proBNP	1.00 (1.00–1.00)	0.804
Peak VO_2_ tertile			MVIC tertile			MP tertile		
High	Reference	‐	High	Reference	‐	High	Reference	‐
Middle	2.85 (0.98–8.31)	0.054	Middle	1.19 (0.40–3.52)	0.752	Middle	1.32 (0.37–4.77)	0.668
Low	6.26 (1.93–20.27)	0.002	Low	3.11 (0.80–12.20)	0.102	Low	5.29 (1.05–26.53)	0.043

Abbreviations: BMI, body mass index; CI, confidence interval; DM, diabetes mellitus; HF, heart failure; HR, hazard ratio; LVEF, left ventricular ejection fraction; MP, muscle power; MVIC, maximum voluntary isomeric contraction; NT‐proBNP. N‐terminal pro b‐type natriuretic peptide; VO_2_, oxygen uptake.

## 4 DISCUSSION

In this study, we found that both MP and MVIC were lower in patients with HF rehospitalization than in those without rehospitalization. Both these parameters also exhibited a significant correlation with the peak VO_2_. However, low MP (and not MVIC) was significantly associated with the risk of rehospitalization for HF in patients with HFrEF.

HF rehospitalization incurs high medical costs, putting a great burden on patients, the health care system, and the social economy. In addition, it may contribute to the long‐term progression of HF and LV dysfunction.^14^ In this study, DM and LVEF are the most significant factors for predicting HF rehospitalization. It is known that HF patients with DM have a higher risk of HF rehospitalization and a worse prognosis than those without DM.^15^ Also, low LVEF is closely associated with cardiovascular outcomes in HF patients.^16^ Despite adjustment of important prognostic factors such as diabetes and LVEF, our study showed that low aerobic exercise capacity and low muscular fitness are significant prognostic factors related to HF rehospitalization.

Peak VO_2_ is the most objective indicator of physical fitness that represents the use of oxygen in the cardiac, circulatory, and respiratory systems and muscles.^17^ CPET is recommended in the 2016 European HF Guidelines for identifying the cause of unexplained dyspnea or for determining the treatment policies.^2^ CPET can provide a more global assessment of patients with HF. CPET parameters are valid prognostic factors for HF, especially peak VO_2_ is closely related to the long‐term prognosis of HF.^7^ In our analysis, among the CPET parameters, peak VO_2_ was most closely associated with to HF rehospitalization (Supplemental Table 2). Our study showed that muscular fitness is also a major predictor of HF prognosis. The isokinetic strength test of the knee flexor muscle showed that the strength index was significantly associated with mortality, although peak VO_2_ was adjusted in multivariable analysis for patients with advanced HF.^6^ Despite the clear association between muscular fitness and long‐term outcomes of HF, muscular fitness is used less frequently than peak VO_2_ for assessing the patients' condition in the clinical settings.

Consistent poor exercise tolerance was observed in the patients with HF and hypothesized to be due to the pathophysiological changes in the skeletal muscle.^18^ Histologic studies on the skeletal muscles in HF identified reduced capillary density and decreased mitochondrial volume.^19^, ^20^ Excessive activation of the sympathetic nervous system and upregulation of the cytokine system induced a decrease in proteins in the muscle and destruction of those proteins, thereby reducing muscle mass.^21^ These muscle‐wasting conditions correlated with maximum peak oxygen uptake in patients with HF.^22^ In our study, muscular fitness and peak VO_2_ were significantly correlated, and patients with low MP, probably those with low peak VO_2_, had poor prognosis for HF. These findings suggested that the association of MP with the prognosis of HF is comparable to that of peak VO_2_. In addition, muscular fitness variables in combination with peak VO_2_ can more reliably predict hospitalization for heart failure. The prognostic ability of the combination of peak VO_2_ and muscular fitness variable was analyzed using the best cut‐off values obtained using ROC analysis. When the peak VO_2_ was low and the MP or MVIC was low, the risk of re‐hospitalization due to HF was 9–20 times higher than that of the high peak VO_2_ and high MP or MVIC (data not shown). However, since the number of subjects is small, further research is needed.

As it is generally recommended to measure peak VO_2_ rather than muscular fitness in evaluating the physical fitness of patients with HF, exercise training is primarily focused on aerobic endurance exercise.^2^ Resistance training is a form of exercise that contracts the muscles against opposing forces that create resistance, overloading the musculoskeletal system to prevent muscle loss and improve the muscle strength.^23^ Although resistance training has survival benefits in patients with HF, increased afterload during the lifting phase in resistance training may adversely affect the LV function and cause negative remodeling.^24^ Therefore, exercise training aimed at improving muscular fitness in patients with HF has not been widely used. Nevertheless, a position statement of the *European Journal of Heart Failure* recommends a patient‐specific strength‐training program relying on the accurate and meticulous evaluation of each patient's physical fitness.^24^ This suggests that there is a need for a reliable method for evaluating muscular fitness that can be safely practiced in patients. However, in several studies, methods for measuring muscular fitness are inconsistent, resulting in a lack of consensus regarding objective representative indicators of muscular fitness. As patients with chronic diseases such as HF have lesser muscular fitness than healthy individuals, it may be difficult to apply general methods of measuring muscular fitness to these patients.

Muscular strength is the force that a muscle or muscle group exerts against resistance in maximal effort. MVIC is a standardized, objective, and sensitive tool for measuring muscle strength.^25^ Power is defined as the product of force and distance divided by the change in time. As a measure of muscular fitness, it can be challenging to separate muscle strength and MP because measuring muscle strength and power is a dynamic process. In our study, the two indicators were closely related. However, when comparing the values of the two indicators as functional measures of muscle performance, MP appeared to be slightly better. In adults aged ≥65 years, decreased MP affects physical performance three times more than decreased muscle strength.^26^ MP decreases with age, possibly occurring earlier than changes in peak muscle strength; this has drawn attention as an essential predictor of reduced activity in older patients.^27^

Only a few studies have measured MP and strength separately in patients with HF; to the best of our knowledge, this is the first study to analyze the long‐term prognostic relationship of these two indicators with HF. We demonstrated that MP was a better indicator of muscular fitness for predicting the long‐term prognosis of HF than MVIC, an indicator of muscle strength. The measurement of MVIC may cause the Valsalva maneuver, which may induce stress in the left ventricle.^24^ As the MP measurement method used in our study is performed according to the individual weight load, it is possible to measure muscular fitness more safely. Therefore, measuring MP as an indicator of prognosis in patients with HF and for guiding muscle training to reduce muscle loss may be useful for facilitating a multidisciplinary approach towards HF.

This study has several limitations. First, our subjects were those who had physical activity sufficient to measure both CPET and muscular fitness. Therefore, there was a high possibility that patients with severe muscle loss were not included, and it might be difficult to generalize the results of this study to all patients with severe advanced HF. Nevertheless, measuring MP will not be difficult in patients with disability because its feasibility has been demonstrated in older populations with sarcopenia. Second, we did not demonstrate that MP was related to the prognosis of HF independently of peak VO_2_. This is because peak VO_2_ and MP were highly correlated and subjects with low peak VO_2_ or low muscular fitness shared clinical features such as old age, female, and high NT‐proBNP levels (Supplemental Table 3–7). However, the fact that MP was significantly associated with the prognosis of HF as much as peak VO_2_ suggests that measuring MP in patients is an alternative to measuring physical fitness. Third, the medications used after discharge were not reflected in the research results. In addition, lower prescription of beta‐blockers at discharge in patients with low MP may have had an impact on long‐term prognosis. Fourth, the number of subjects who participated in the study was relatively small.

In conclusion, aerobic exercise capacity and muscle fitness were associated with the prognosis of patients with HFrEF. Compared with MVIC, which is the traditional method of measuring knee extensor fitness, measurement of MP was found to be a better predictor for HF rehospitalization in these patients.

## CONFLICT OF INTEREST

The authors declare that they have no competing interests.

## Supporting information


**Supplemental figure 1** Aerobic exercise capacity according to tertile of maximal voluntary isometric contraction (A), and muscle power (B).

**Supplemental figure 2** ROC analysis of peak VO2 (A), maximal voluntary isometric contraction (B), and muscle power (C) for prediction of heart failure rehospitalization

**Supplemental table 1** Heart failure rehospitalization of the study population grouped by the tertile of aerobic exercise capacity and muscle fitness
**Supplemental Table 2**. Multivariable Cox proportional hazard regression analyses of cardiopulmonary exercise test variables for HF rehospitalization
**Supplemental Table 3**. Baseline characteristics according to the tertile of peak VO_2_
**Supplemental Table 4**. Baseline characteristics according to the tertile of maximal voluntary isometric contraction
**Supplemental Table 5**. Baseline characteristics according to the tertile of muscle power
**Supplemental Table 6**. Baseline characteristics according to cut‐off value of maximal voluntary isometric contraction
**Supplemental Table 7**. Baseline characteristics according to cut‐off value of muscle power

**Supplemental video file 1**. Assessment of maximal voluntary isometric contraction.

**Supplemental video file 2**. Assessment of muscle power.

## Data Availability

The data that support the findings of this study are available from the corresponding author upon reasonable request.
