# Supplementary figures and images for: Association of muscular fitness with rehospitalization for heart failure with reduced ejection fraction
Source: Clin Cardiol. 2020 Dec 25;44(2):244–51. doi: 10.1002/clc.23535 (PMC7852176; doi:10.1002/clc.23535)

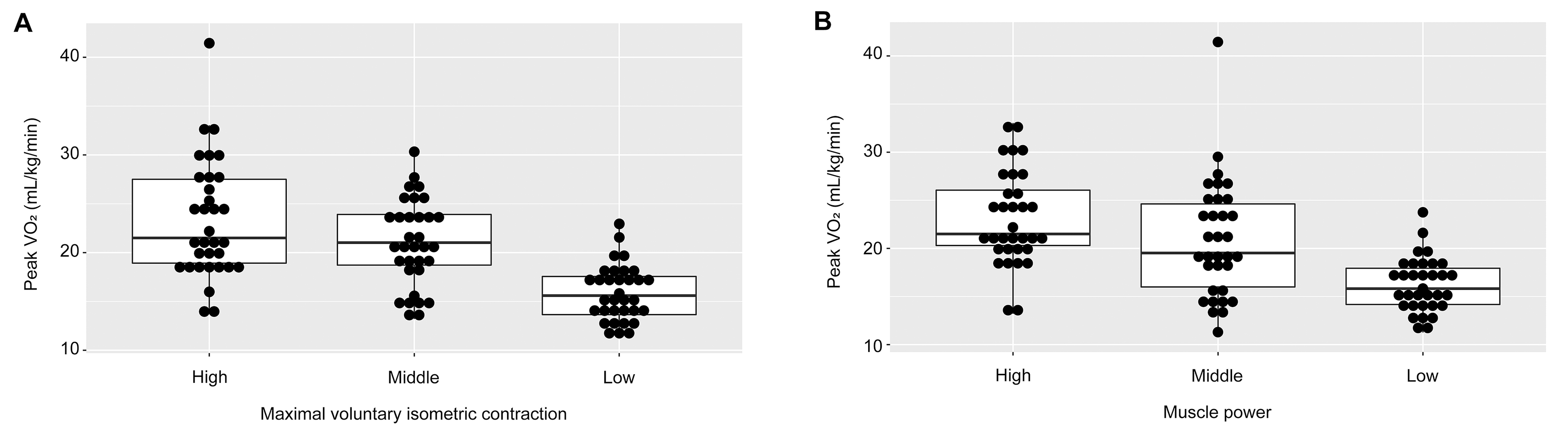

Supplement: Supplementary file 1 — Supplemental figure 1 Aerobic exercise capacity according to tertile of maximal voluntary isometric contraction (A), and muscle power (B). [file CLC-44-244-s001.jpg]

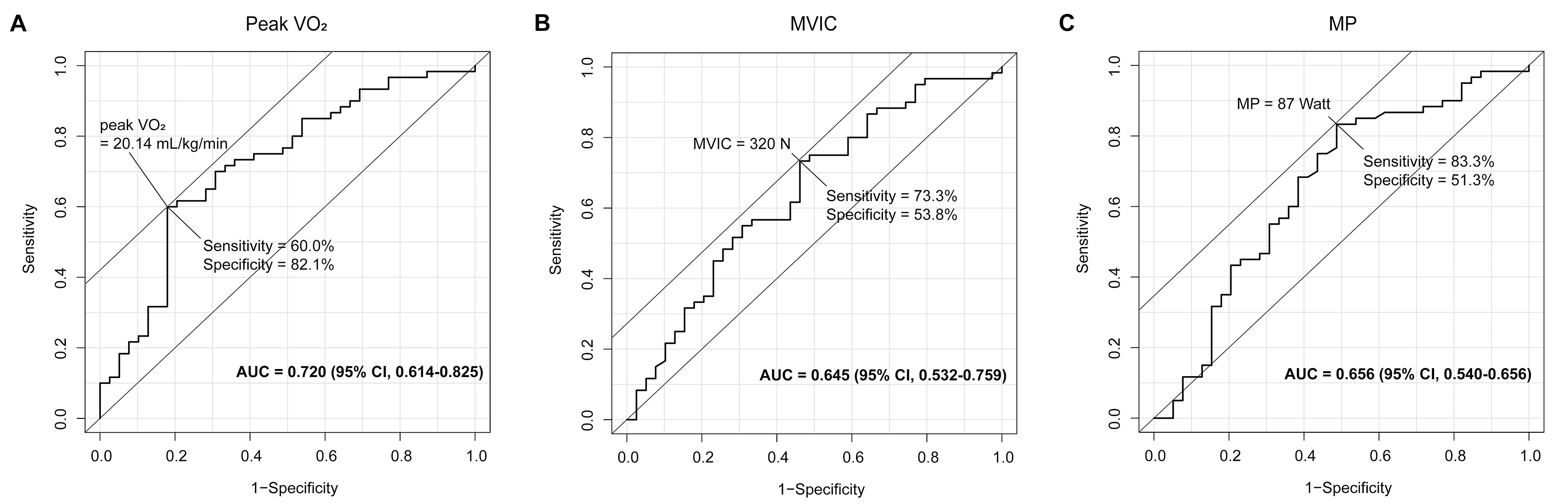

Supplement: Supplementary file 2 — Supplemental figure 2 ROC analysis of peak VO2 (A), maximal voluntary isometric contraction (B), and muscle power (C) for prediction of heart failure rehospitalization [file CLC-44-244-s002.jpg]
