# Supplementary material for: Association of muscular fitness with rehospitalization for heart failure with reduced ejection fraction
Source: Clin Cardiol. 2020 Dec 25;44(2):244–51. doi: 10.1002/clc.23535 (PMC7852176; doi:10.1002/clc.23535)
Supplement: Supplementary file 3 — Supplemental table 1 Heart failure rehospitalization of the study population grouped by the tertile of aerobic exercise capacity and muscle fitness Supplemental Table 2. Multivariable Cox proportional hazard regression analyses of cardiopulmonary exercise test variables for HF rehospitalization Supplemental Table 3. Baseline characteristics according to the tertile of peak VO2 Supplemental Table 4. Baseline characteristics according to the tertile of maximal voluntary isometric contraction Supplemental Table 5. Baseline characteristics according to the tertile of muscle power Supplemental Table 6. Baseline characteristics according to cut‐off value of maximal voluntary isometric contraction Supplemental Table 7. Baseline characteristics according to cut‐off value of muscle power [file CLC-44-244-s003.docx]

Supplemental table 1. Heart failure rehospitalization of the study population grouped by the tertile of aerobic exercise capacity and muscle fitness

|  | High  (N=33) | Middle  (N=33) | Low  (N=33) | P-value |
| --- | --- | --- | --- | --- |
| Peak VO_2_, ml/kg/min | ≥21.5 | 17.2-<21.5 | <17.2 |  |
| HF rehospitalization, N(%) | 7 (21.2) | 13 (39.4) | 19 (57.6) | 0.010 |
| MVIC, N | ≥440 | 309-<440 | <309 |  |
| HF rehospitalization, N(%) | 9 (27.3) | 12 (36.4) | 18 (54.5) | 0.070 |
| MP, Watt | ≥154.5 | 91.9-<154.5 | <91.9 |  |
| HF rehospitalization, N(%) | 8 (24.2) | 11 (33.3) | 20 (60.6) | 0.007 |
| HF, heart failure; MP, muscle power; MVIC, maximum voluntary isomeric contraction; VO_2_, oxygen uptake | | | | |

Supplemental table 2. Multivariable Cox proportional hazard regression analyses of cardiopulmonary exercise test variables for HF rehospitalization

|  | HR (95% CI) | P-value |
| --- | --- | --- |
| Peak VO_2_ | 0.828 (0.743-0.925) | <0.001 |
| Exercise time | 0.996 (0.994-0.999) | 0.002 |
| VE/VCO_2_ slope | 1.038 (0.997-1.080) | 0.067 |
| HR reserve | 0.984 (0.966-1.003) | 0.097 |
| PetCO_2_ | 0.991 (0.934-1.053) | 0.773 |
| AT | 0.958 (0.903-1.017) | 0.158 |
| Multivariable model adjusted for age, sex, body mass index, diabetes mellitus, and left ventricular ejection fraction, and N-Terminal Pro-B-type natriuretic peptide.  AT, anaerobic threshold; HRR, heart rate reserve; PetCO_2,_ pulmonary end-tidal CO_2_; VCO_2_, carbon dioxide production; VE, ventilatory equivalents; VO_2_, oxygen uptake | | |

Supplemental table 3. Baseline characteristics according to the tertile of peak VO_2_

|  | High | Middle | Low | p |
| --- | --- | --- | --- | --- |
|  | (N=33) | (N=33) | (N=33) |  |
| *Demographic findings* |  |  |  |  |
| Age, years | 55.2 ± 13.5 | 58.8 ± 12.5 | 61.0 ± 13.7 | 0.199 |
| Men, N(%) | 28 (84.8%) | 24 (72.7%) | 12 (36.4%) | <0.001 |
| Height, cm | 166.4 ± 8.7 | 165.0 ± 8.1 | 160.2 ± 8.8 | 0.010 |
| Weight, kg | 68.2 ± 11.0 | 67.0 ± 17.6 | 58.0 ± 12.8 | 0.008 |
| BMI, kg/m^2^ | 24.6 ± 3.4 | 24.3 ± 4.7 | 22.5 ± 3.9 | 0.070 |
| LV ejection fraction, % | 27.4 ± 9.0 | 27.8 ± 7.5 | 27.3 ± 8.1 | 0.967 |
| Sinus rhythm, N(%) | 27 (81.8%) | 22 (66.7%) | 24 (72.7%) | 0.371 |
| DM, N(%) | 7 (25.0%) | 10 (33.3%) | 8 (25.0%) | 0.707 |
| Hypertension, N(%) | 12 (42.9%) | 14 (46.7%) | 13 (40.6%) | 0.890 |
| Ischemic cardiomyopathy, N(%) | 11 (33.3%) | 13 (39.4%) | 8 (24.2%) | 0.416 |
| *Laboratory findings* |  |  |  |  |
| BUN, mg/dL | 17.0 ± 5.2 | 19.7 ± 7.9 | 18.8 ± 6.4 | 0.254 |
| Creatinine, mg/dL | 0.9 ± 0.2 | 1.0 ± 0.5 | 0.8 ± 0.3 | 0.122 |
| Serum total protein, d/dL | 6.8 ± 0.6 | 6.9 ± 0.6 | 6.5 ± 0.7 | 0.014 |
| Serum albumin, d/dL | 4.1 ± 0.4 | 4.0 ± 0.3 | 3.8 ± 0.5 | 0.077 |
| Na^+^, mmol/L | 140.7 ± 2.1 | 140.0 ± 3.0 | 139.2 ± 2.6 | 0.074 |
| K^+^, mmol/L | 4.4 ± 0.4 | 4.5 ± 0.5 | 4.4 ± 0.5 | 0.620 |
| NT-proBNP, pg/mL | 898.5 (417.0-1623.5) | 1331.0 (680.0-1833.0) | 1968.0 (1084.5-3116.0) | 0.002 |
| *Medications at discharge* |  |  |  |  |
| ACE inhibitor, N(%) | 15 (45.5%) | 16 (48.5%) | 11 (33.3%) | 0.420 |
| ARB, N(%) | 11 (33.3%) | 11 (33.3%) | 14 (42.4%) | 0.675 |
| Beta blocker, N(%) | 33 (100.0%) | 24 (72.7%) | 18 (54.5%) | <0.001 |
| Ivabradine, N(%) | 2 ( 6.1%) | 1 ( 3.0%) | 7 (21.2%) | 0.032 |
| Loop diuretics, N(%) | 20 (60.6%) | 26 (78.8%) | 28 (84.8%) | 0.062 |
| MRA, N(%) | 19 (57.6%) | 28 (84.8%) | 27 (81.8%) | 0.020 |
| Data is presented as mean ± SD, N(%) or median (IQR). ACE, angiotensin converting enzyme; ARB, angiotensin receptor blocker; BMI, body mass index; BUN, blood urea nitrogen; DM, diabetes mellitus; LV, left ventricle; CMP; MRA, mineralocorticoid antagonist. | | | | |

Supplemental table 4. Baseline characteristics according to the tertile of maximal voluntary isometric contraction

|  | High | Middle | Low | p |
| --- | --- | --- | --- | --- |
|  | (N=33) | (N=33) | (N=33) |  |
| *Demographic findings* |  |  |  |  |
| Age, years | 53.4 ± 13.6 | 56.2 ± 13.4 | 65.3 ± 10.1 | <0.001 |
| Men, N(%) | 33 (100.0%) | 22 (66.7%) | 9 (27.3%) | <0.001 |
| Height, cm | 169.1 ± 5.8 | 165.0 ± 8.7 | 157.5 ± 7.8 | <0.001 |
| Weight, kg | 74.8 ± 15.0 | 65.1 ± 10.5 | 53.4 ± 9.1 | <0.001 |
| BMI, kg/m^2^ | 26.0 ± 4.2 | 23.9 ± 3.5 | 21.5 ± 3.3 | <0.001 |
| LV ejection fraction, % | 27.1 ± 8.6 | 26.3 ± 8.1 | 29.2 ± 7.7 | 0.334 |
| Sinus rhythm, N(%) | 26 (78.8%) | 26 (78.8%) | 21 (63.6%) | 0.271 |
| DM, N(%) | 5 (20.0%) | 11 (34.4%) | 9 (27.3%) | 0.484 |
| Hypertension, N(%) | 9 (36.0%) | 13 (40.6%) | 17 (51.5%) | 0.462 |
| Ischemic cardiomyopathy, N(%) | 12 (36.4%) | 11 (33.3%) | 9 (27.3%) | 0.724 |
| *Laboratory findings* |  |  |  |  |
| BUN, mg/dL | 18.1 ± 6.1 | 17.7 ± 4.5 | 19.8 ± 8.6 | 0.368 |
| Creatinine, mg/dL | 1.0 ± 0.2 | 0.9 ± 0.2 | 0.9 ± 0.6 | 0.678 |
| Serum total protein, d/dL | 6.8 ± 0.7 | 6.7 ± 0.6 | 6.6 ± 0.7 | 0.404 |
| Serum albumin, d/dL | 4.1 ± 0.4 | 3.9 ± 0.4 | 3.9 ± 0.5 | 0.086 |
| Na^+^, mmol/L | 140.4 ± 2.4 | 140.0 ± 2.3 | 139.6 ± 3.2 | 0.460 |
| K^+^, mmol/L | 4.5 ± 0.4 | 4.3 ± 0.4 | 4.5 ± 0.6 | 0.146 |
| NT-proBNP, pg/mL | 689.0 (309.5-1623.5) | 1444.0 (900.5-1929.0) | 1814.0 (1151.0-3008.5) | 0.001 |
| *Medications at discharge* |  |  |  |  |
| ACE inhibitor, N(%) | 15 (45.5%) | 16 (48.5%) | 11 (33.3%) | 0.420 |
| ARB, N(%) | 14 (42.4%) | 9 (27.3%) | 13 (39.4%) | 0.400 |
| Beta blocker, N(%) | 29 (87.9%) | 25 (75.8%) | 21 (63.6%) | 0.071 |
| Ivabradine, N(%) | 5 (15.2%) | 3 ( 9.1%) | 2 ( 6.1%) | 0.459 |
| Loop diuretics, N(%) | 23 (69.7%) | 24 (72.7%) | 27 (81.8%) | 0.499 |
| MRA, N(%) | 22 (66.7%) | 27 (81.8%) | 25 (75.8%) | 0.362 |
| Data is presented as mean ± SD, N(%) or median (IQR). ACE, angiotensin converting enzyme; ARB, angiotensin receptor blocker; BMI, body mass index; BUN, blood urea nitrogen; DM, diabetes mellitus; LV, left ventricle; CMP; MRA, mineralocorticoid antagonist. | | | | |

Supplemental table 5. Baseline characteristics according to the tertile of muscle power

|  | High | Middle | Low | p |
| --- | --- | --- | --- | --- |
|  | (N=33) | (N=33) | (N=33) |  |
| *Demographic findings* |  |  |  |  |
| Age, years | 50.6 ± 11.8 | 60.1 ± 14.5 | 64.2 ± 9.7 | <0.001 |
| Men, N(%) | 33 (100.0%) | 23 (69.7%) | 8 (24.2%) | <0.001 |
| Height, cm | 169.8 ± 6.1 | 164.1 ± 8.1 | 157.7 ± 7.8 | <0.001 |
| Weight, kg | 75.3 ± 14.6 | 65.5 ± 9.5 | 52.4 ± 9.1 | <0.001 |
| BMI, kg/m^2^ | 26.0 ± 4.2 | 24.3 ± 3.1 | 21.1 ± 3.3 | <0.001 |
| LV ejection fraction, % | 27.8 ± 8.8 | 26.2 ± 7.9 | 28.5 ± 7.8 | 0.504 |
| Sinus rhythm, N(%) | 25 (75.8%) | 27 (81.8%) | 21 (63.6%) | 0.232 |
| DM, N(%) | 4 (16.7%) | 8 (24.2%) | 13 (39.4%) | 0.142 |
| Hypertension, N(%) | 9 (37.5%) | 14 (42.4%) | 16 (48.5%) | 0.705 |
| Ischemic cardiomyopathy, N(%) | 14 (42.4%) | 9 (27.3%) | 9 (27.3%) | 0.315 |
| *Laboratory findings* |  |  |  |  |
| BUN, mg/dL | 17.7 ± 5.8 | 18.3 ± 4.9 | 19.5 ± 8.6 | 0.533 |
| Creatinine, mg/dL | 1.0 ± 0.2 | 0.9 ± 0.2 | 0.9 ± 0.6 | 0.879 |
| Serum total protein, d/dL | 6.8 ± 0.6 | 6.8 ± 0.6 | 6.6 ± 0.7 | 0.332 |
| Serum albumin, d/dL | 4.1 ± 0.4 | 4.0 ± 0.4 | 3.8 ± 0.5 | 0.067 |
| Na^+^, mmol/L | 140.2 ± 2.5 | 140.7 ± 1.7 | 139.0 ± 3.3 | 0.032 |
| K^+^, mmol/L | 4.5 ± 0.4 | 4.4 ± 0.5 | 4.5 ± 0.6 | 0.835 |
| NT-proBNP, pg/mL | 904.0 (353.0-1775.5) | 1389.5 (662.0-1799.5) | 1814.0 (1213.5-3847.5) | 0.001 |
| *Medications at discharge* |  |  |  |  |
| ACE inhibitor, N(%) | 13 (39.4%) | 17 (51.5%) | 12 (36.4%) | 0.420 |
| ARB, N(%) | 14 (42.4%) | 10 (30.3%) | 12 (36.4%) | 0.592 |
| Beta blocker, N(%) | 27 (81.8%) | 28 (84.8%) | 20 (60.6%) | 0.043 |
| Ivabradine, N(%) | 4 (12.1%) | 4 (12.1%) | 2 ( 6.1%) | 0.641 |
| Loop diuretics, N(%) | 22 (66.7%) | 26 (78.8%) | 26 (78.8%) | 0.316 |
| MRA, N(%) | 24 (72.7%) | 24 (72.7%) | 26 (78.8%) | 0.807 |
| Data is presented as mean ± SD, N(%) or median (IQR). ACE, angiotensin converting enzyme; ARB, angiotensin receptor blocker; BMI, body mass index; BUN, blood urea nitrogen; DM, diabetes mellitus; LV, left ventricle; CMP; MRA, mineralocorticoid antagonist. | | | | |

Supplemental table 6. Baseline characteristics according to cut-off value of maximal voluntary isometric contraction

|  | MVIC ≥320 N | MVIC <320 N | | P value |
| --- | --- | --- | --- | --- |
|  | (N=62) | (N=37) | |  |
| *Demographic findings* |  |  | |  |
| Age, years | 55.5 ± 13.3 | 63.0 ± 12.1 | | 0.006 |
| Men, N(%) | 54 (87.1%) | 10 (27.0%) | | <0.001 |
| Height, cm | 167.2 ± 7.6 | 158.3 ± 8.0 | | <0.001 |
| Weight, kg | 70.7 ± 13.8 | 53.9 ± 9.2 | | <0.001 |
| BMI, kg/m^2^ | 25.2 ± 4.0 | 21.5 ± 3.1 | | <0.001 |
| LV ejection fraction, % | 26.8 ± 8.3 | 28.7 ± 7.9 | | 0.276 |
| Sinus rhythm, N(%) | 49 (79.0%) | 24 (64.9%) | | 0.189 |
| DM, N(%) | 15 (28.3%) | 10 (27.0%) | | 0.999 |
| Hypertension, N(%) | 22 (41.5%) | 17 (45.9%) | | 0.840 |
| Ischemic cardiomyopathy, N(%) | 22 (35.5%) | 10 (27.0%) | | 0.517 |
| *Laboratory findings* |  | |  |  |
| BUN, mg/dL | 17.9 ± 5.2 | 19.5 ± 8.5 | | 0.329 |
| Creatinine, mg/dL | 0.9 ± 0.2 | 0.9 ± 0.5 | | 0.705 |
| Serum total protein, d/dL | 6.8 ± 0.6 | 6.6 ± 0.6 | | 0.096 |
| Serum albumin, d/dL | 4.1 ± 0.4 | 3.9 ± 0.5 | | 0.043 |
| Na^+^, mmol/L | 140.2 ± 2.4 | 139.6 ± 3.1 | | 0.301 |
| K^+^, mmol/L | 4.4 ± 0.4 | 4.4 ± 0.6 | | 0.937 |
| NT-proBNP, pg/mL | 1060.0 (485.0-1744.5) | 1814.0 (1084.5-3116.0) | | 0.001 |
| *Medications at discharge* |  |  | |  |
| ACE inhibitor, N(%) | 28 (45.2%) | 14 (37.8%) | | 0.615 |
| ARB, N(%) | 22 (35.5%) | 14 (37.8%) | | 0.984 |
| Beta blocker, N(%) | 53 (85.5%) | 22 (59.5%) | | 0.007 |
| Ivabradine, N(%) | 7 (11.3%) | 3 (8.1%) | | 0.87 |
| Loop diuretics, N(%) | 44 (71.0%) | 30 (81.1%) | | 0.378 |
| MRA, N(%) | 46 (74.2%) | 28 (75.7%) | | 0.999 |
| Data is presented as mean ± SD, N(%) or median (IQR). ACE, angiotensin converting enzyme; ARB, angiotensin receptor blocker; BMI, body mass index; BUN, blood urea nitrogen; DM, diabetes mellitus; LV, left ventricle; CMP; MRA, mineralocorticoid antagonist. | | | | |

Supplemental table 7. Baseline characteristics according to cut-off value of muscle power

|  | MP ≥87 Watt | MP <87 Watt | | P value |
| --- | --- | --- | --- | --- |
|  | (N=69) | (N=30) | |  |
| *Demographic findings* |  |  | |  |
| Age, years | 55.8 ± 13.9 | 64.1 ± 10.0 | | 0.004 |
| Men, N(%) | 57 (82.6%) | 7 (23.3%) | | <0.001 |
| Height, cm | 166.6 ± 7.9 | 157.6 ± 7.8 | | <0.001 |
| Weight, kg | 69.9 ± 13.2 | 51.9 ± 9.2 | | <0.001 |
| BMI, kg/m^2^ | 25.1 ± 3.7 | 20.9 ± 3.4 | | <0.001 |
| LV ejection fraction, % | 27.2 ± 8.5 | 28.3 ± 7.4 | | 0.548 |
| Sinus rhythm, N(%) | 53 (76.8%) | 20 (66.7%) | | 0.420 |
| DM, N(%) | 13 (21.7%) | 12 (40.0%) | | 0.114 |
| Hypertension, N(%) | 25 (41.7%) | 14 (46.7%) | | 0.821 |
| Ischemic cardiomyopathy, N(%) | 24 (34.8%) | 8 (26.7%) | | 0.576 |
| *Laboratory findings* |  | |  |  |
| BUN, mg/dL | 17.8 ± 5.3 | 20.2 ± 8.7 | | 0.171 |
| Creatinine, mg/dL | 0.9 ± 0.2 | 0.9 ± 0.6 | | 0.819 |
| Serum total protein, d/dL | 6.8 ± 0.6 | 6.7 ± 0.6 | | 0.449 |
| Serum albumin, d/dL | 4.0 ± 0.4 | 3.9 ± 0.5 | | 0.078 |
| Na^+^, mmol/L | 140.4 ± 2.2 | 139.0 ± 3.3 | | 0.035 |
| K^+^, mmol/L | 4.4 ± 0.4 | 4.5 ± 0.6 | | 0.440 |
| NT-proBNP, pg/mL | 1133.0 (500.0-1812.0) | 1866.5 (1213.5-4591.0) | | <0.001 |
| *Medications at discharge* |  |  | |  |
| ACE inhibitor, N(%) | 31 (44.9%) | 11 (36.7%) | | 0.587 |
| ARB, N(%) | 26 (37.7%) | 10 (33.3%) | | 0.852 |
| Beta blocker, N(%) | 57 (82.6%) | 18 (60.0%) | | 0.031 |
| Ivabradine, N(%) | 8 (11.6%) | 2 (6.7%) | | 0.700 |
| Loop diuretics, N(%) | 49 (71.0%) | 25 (83.3%) | | 0.296 |
| MRA, N(%) | 50 (72.5%) | 24 (80.0%) | | 0.588 |
| Data is presented as mean ± SD, N(%) or median (IQR). ACE, angiotensin converting enzyme; ARB, angiotensin receptor blocker; BMI, body mass index; BUN, blood urea nitrogen; DM, diabetes mellitus; LV, left ventricle; CMP; MRA, mineralocorticoid antagonist. | | | | |
